# Change in China’s SRB: A Dynamic Spatial Panel Approach

**DOI:** 10.3390/ijerph17218018

**Published:** 2020-10-30

**Authors:** Tingshuai Ge, Li Mei, Xiujun Tai, Quanbao Jiang

**Affiliations:** 1 Institute for Population and Development Studies, Xi’an Jiaotong University, Xi’an 710049, China; bacca-gts@stu.xjtu.edu.cn (T.G.); meili1246@stu.xjtu.edu.cn (L.M.); 2 School of Economics and Management, Shanxi Normal University, Linfen 041004, China; taixj@sxnu.edu.cn

**Keywords:** sex ratio at birth, spatial dependence, spatial Durbin model, China

## Abstract

There has been some research on factors affecting China’s rising sex ratio at birth (SRB), but the spatial dependence has been largely neglected. With China’s census and sample survey data and the dynamic spatial Durbin model; we analyzed the changes in SRB in China. We found that SRB and its influencing factors were spatially correlated at the provincial level. For direct effects; urbanization significantly reduced SRB in this region; while strict family planning policies increased SRB in the local region. For indirect effects; the increase in per capita Gross Domestic Product and urbanization led to an increase in the SRB of the neighboring regions through population mobility. By comparison; educational improvement in one region benefited the neighboring provinces and reduced SRB.

## 1. Introduction

Since the 1980s, the sex ratio at birth (the ratio of live male births to live female births, generally expressed as the number of live male births for every 100 live female births, hereafter SRB) in China has continued to rise. China’s census data show that the SRB was 108.5 in 1982 and 111.4 in 1990; it rose to 119.9 in 2000; and reached 121.2 in 2010 [1,2,3,4]. After 2010, the SRB has been declining, but it is still much higher than normal. The long-term imbalance in the SRB has led to a large number of missing girls [5], exerted a far-reaching and profound effect on the long-term development of the Chinese population [6], the revival of sex industry and the spread of sexually transmitted diseases [7,8], economic behavior [9,10], and other aspects of demographic and socioeconomic development of China.

Some studies have analyzed the causes of the imbalance in SRB in China from the perspective of economics [11,12,13], society [14,15], culture [13,16], and social policies [11,17], and reached many meaningful conclusions. Recently, the physical environments, including air quality and ambient temperatures, were also found to have an influence on SRB [18,19]. However, the spatial dependence of the SRB has never been considered in the existing studies on SRB. There are more than thirty administrative regions within China at the provincial level. Each administrative region has its own social, economic, cultural, and physical environmental characteristics, as well as local family planning policies [20], resulting in vast differences between them.

The change in the SRB is spatially clustered and diffused, making one or more regions have a high SRB, and spreading to the surrounding regions [21,22]. This spatial clustering characteristic of SRB became more significant between 2000 and 2010 [23]. In today’s increasingly convenient transportation and faster dissemination of information, the concept of fertility will continue to spread to neighboring regions, affecting the gender choices of individuals and the SRB [23]. Influenced by economic, social, cultural, policy, and physical environmental factors in neighboring regions, the SRB may also converge in neighboring regions [24]. The use of traditional statistical methods without fully considering the spatial dependence of the SRB through the use of spatial analysis methods will affect the research results quite significantly.

Using China’s census and sample survey data, and the dynamic spatial Durbin model (hereafter SDM), this article examined the impact of economic, social, cultural, policy, and physical environmental factors on SRB. This study adds to the literature in the following aspects: firstly, compared with traditional statistical methods, we used a spatial econometric model to examine the spatial clustering effects on SRB estimation. Secondly, this paper used dynamic SDM. Considering the endogenous interaction effects, the exogenous interaction effects, and the time-lagged SRB, the dynamic SDM can illustrate more accurately the mechanism of spatial effects, the short- and long-term effects of each explanatory variable on SRB. The rest of this paper is organized as following. In the following section, we review factors that affect the SRB and its spatial dependence. The third section describe the data and methods, including the composition of dynamic SDM and the decomposition of direct and indirect effects, followed by the section on results. In the last section, we discuss the findings of this paper.

## 2. Literature Review

Changes in SRB are the result of socioeconomic, cultural, and policy environments, including economic development, urbanization level, education level, family planning policies, and physical environments, including air quality and ambient temperatures. There are also some studies involving spatial analysis of SRB.

### 2.1. Provincial Differences in China

China is characterized by vast provincial differences in terms of socioeconomic, cultural, policy, and physical environments. With regard to socioeconomic environments, since the economic reform and open up, the socioeconomic development of China’s provinces has begun to show large differences, with provinces in the east of China have the highest degree of economic growth and urbanization level, followed by the provinces in the central region, and finally the provinces in the western region. Similarly, the cultural environments vary markedly from province to province. The patrilineality, patriarchy, and patrilocality in China have led to strong son preference, with people in the south China have the strongest son preference, and people in the Yangtze River Basin and Northeast China have a weaker preference for boys [25]. The policy environments also vary by province. China’s family planning policy, which has led to regional SRB disparities, has been decentralized by region. Around 2000, six provinces implemented a one-child policy for both urban and rural couples among China’s 30 mainland provinces (excluding Tibet); five provinces implemented a two-child policy; and the other 19 provinces implemented a 1.5-child policy, meaning that a second birth was permitted for rural couples whose first child was a daughter, while urban couples were subject to a one-child police policy [20]. For physical environments, the vast land area and huge economic development differences make the physical environment of China’s provinces also have great differences. It shows that the environmental quality of provinces in the eastern, southern, and coastal region is better than the environmental quality of provinces in the northern inland region [26].

### 2.2. Previous Studies Using Nonspatial Methods

#### 2.2.1. Economic Development and SRB

The relationship between the level of economic development and SRB is controversial. Some studies suggest that there is no statistically significant relationship between economic growth and SRB. Studies of South Korea and Taiwan [11] and studies using the 2000 and previous China’s census and sample survey data [27] did not find a significant correlation between economic levels and SRB. Some studies suggest that economic development has restrained the rise in SRB. The economic development has reduced the significance of sons in providing labor and social security, weakened son preferences, and subsequently reduced SRB; it can also improve people’s gender perceptions and curb the increase in SRB [28]. Chinese studies have also found that improvements in regional socioeconomic conditions can alleviate the imbalance in SRB in China [29]. However, some literature points out that economic development and high income increases the possibility of realizing son preferences, leading to an increase in SRB [30].

Some other studies have suggested that the relationship between economic development and SRB is related to the stage of development. Guilmoto and Ren [12] used China’s 2000 census data and 2005 one percent population sample survey data, and found that there is an inverted U-shaped relationship between economic level and SRB, that is, lower SRB appeared in poor and wealthy families. Yang and Li [13] pointed out that only when the level of economic development reaches a certain critical point (per capita GDP—Gross Domestic Product reaches 40,000 Yuan, around $5700 USD) will SRB have a curtailing effect. A GDP per capita below the critical point promoted the rise of SRB.

#### 2.2.2. Urbanization Level and SRB

Some studies have found that urbanization can curb the rise in SRB. China’s rural–urban migration and rapid urbanization, while propelling economic and social development, are continually changing people’s lifestyles, fertility ideology and behaviors [31]. Urbanization has accelerated the transfer of rural population to urban regions, and population migration has changed the system, environment, and culture on which people depend, and brought about a change in fertility ideology and gender perception, thereby reducing SRB [32]. Population mobility and migration will weaken the son preference, and the increasing urbanization and industrialization will have a more significant impact on family fertility decision-making than only the rising per capita GDP [15].

Studies have also suggested that a large number of immigrants have led to an increase in urban SRB. An analysis of data from Beijing found that the high SRB of immigrants was the leading cause of the imbalance in SRB [14]. In the process of cultural adaptation of rural migrants, the preference for sons is not significantly weakened over time. Their SRB is not substantially different from that of rural non-migrants, which exacerbates high SRB in urban regions [33].

#### 2.2.3. Education Level and SRB

Education level, as a major indicator of social development, is considered an essential factor in reducing gender inequality [34]. Some studies suggest that more educated women are more inclined to the ideology of gender equality and that the increase in women’s education can help curb the rise in SRB [35]. To increase the education level is regarded as a critical way to lower son preference and to curtail the increase in SRB. However, the relationship between China’s education level and SRB is still controversial.

Some studies have suggested that an increase in education level reduces the SRB [36]. Data from China’s 2005 one percent population sample survey showed that the higher the educational attainment of mothers, the lower the sex ratio of children born [37]. However, some other studies argue that the increase in education does not inhibit the increase in SRB [27]. With China’s 1990 census data, Guilmoto [16] confirmed that there was a significant positive relationship between education level and SRB. Still other studies have suggested that the relationship between education and SRB is an inverted U-shape. An inverted U-shaped relationship was reported in the group comparison by the education level of mothers using China’s 1990 census data [11]. Yang and Li [13] also confirmed this inverted “U” relationship using data from 1990 to 2010, and further pointed out that education will only inhibit the SRB when the average schooling is nine years and above; otherwise, improvements in schooling will be used as an instrument to realize son preference and reach the ideal children gender structure.

#### 2.2.4. Family Planning Policies and SRB

The relationship between China’s family planning policies and SRB is also controversial. Some studies have suggested that the increase in SRB is not related to family planning policies [11,17]. The 2000 census data showed that SRB in rural regions with relatively loose fertility policies and ethnic minorities was high, and SRB in provinces with strict family planning policies was not so high, which indicates that SRB is not correlated with the restrictive policy [38]. Nevertheless, more research suggests that strict family planning policies have led to an increase in the SRB [39]. Strict family planning policies accounted for 94%, 57%, and 54% of changes in SRB in the 1980s, 1990s, and 2001–2005, respectively, and were the main factors leading to the high SRB [40].

#### 2.2.5. Air Quality and SRB

China has been paying a high environmental price, such as air contamination, for economic development since the economic reform and open up [41,42]. Previous studies have pointed out that long-term exposure to sulfur dioxide may alter the SRB, but the results are controversial. Many studies have pointed out that long-term exposure to poorer air quality will decrease SRB [43], while some studies have found that poorer air quality will increase SRB [19]. There are also some studies that have not found a significant relationship between them [44,45]. Some scholars suggest that the relationship between air quality and SRB may change with exposure to different chemicals and duration of exposure [46].

#### 2.2.6. Ambient Temperatures and SRB

Since the mid-1970s, the global average temperature has risen by 0.5 °C, and it is predicted to continue to rise by 1.4–5.8 °C by 2100 [47]. Previous studies have examined the impact of climate warming on SRB, but the results are quite controversial. Using the 45-year-long data in northern Finland, Helle et al. [18] found that more males are born in warmer years, with one-degree Celsius increase in average temperature corresponding to about 1% more sons each year. However, other study suggested that more females are born in warmer periods [48]. Besides, there are some studies that did not detect the significant relationship between ambient temperatures and SRB [49].

### 2.3. Spatial Dependence of SRB

From the above review, it can be seen that the existing literature on factors of SRB includes economic development, urbanization level, education level, family planning policies, air quality, and ambient temperatures. But the spatial dependence of these factors has been mostly neglected, which may cause bias in the estimation results.

Some studies have pointed out that SRB is spatially clustered. Using county-level data from China’s 1990 and 2000 censuses, some studies have found significant spatial clustering of sex ratios among children aged 0–4 [16,50]. China’s 1982 to 2000 census data showed marked regional differences in the sex ratio of children aged 0–14 years old [27]. SRB is significantly spatial clustered at the county level [20], and at the provincial level [23].

Given significant spatial dependence of SRB in China, the spatial effect should be considered in examining China’s SRB. Lavely and Cai [51] used county-level data from the 2000 census, and found spatial clustering of sex ratio among children aged 0–4 years. They emphasized the existence of spatial dependence and the application of spatial econometric models when studying the sex ratio of children in China. Using the spatial lag model and spatial error model and China’s 2000 provincial census data, Nie and Sun [52] found that education has a smaller direct impact on SRB, but it indirectly affects SRB through factors such as spatial geography, economics, and family planning policies. The explanatory power of the regression model analyzing factors influencing the SRB at the prefectural level is dramatically improved when the spatial clustering effect is included in the spatial lag and spatial error models [24]. Compared with the Ordinary Least Squares (OLS) model, the spatial model better fits the relationship between explanatory variables and SRB [21].

### 2.4. Spatial Dependence of Explanatory Variables

Factors that affect SRB, such as per capita GDP, urbanization level, education level, air quality, and ambient temperatures are spatially clustered as well. With a provincial dataset for 1978–2003 [53] and another set for 1978–2012 [54], it was found that the per capita GDP was spatially clustered, and the degree of spatial dependence has been increasing annually. Analysis of the prefecture-level data from China’s 2010 censuses found a significant positive spatial autocorrelation in the urbanization rate. The mobility of labor between different cities played a vital role in the formation of spatial clustering in urbanization [55]. Using provincial-level data from 1996 to 2013, Fang and Luo [56] found a significant positive spatial autocorrelation in human capital measured in educational attainment. Furthermore, air quality and ambient temperatures were also found to be spatially clustered in previous studies [42,57].

The above analysis shows that there are many studies on factors affecting SRB in China, but the impact of economic development, urbanization level, education level, family planning policies, air quality, and ambient temperatures on the size and direction of SRB is quite controversial. In terms of methods, existing studies mostly used qualitative analysis and traditional statistical methods. The few studies employing spatial analysis mainly used spatial lag models or spatial error models to analyze cross-sectional data. Both the SRB itself, and the economic, social, cultural, policy, and physical environmental factors that affect the change in SRB are strongly spatially correlated. If the spatial dependence of these factors is not included in the model or included in an inappropriate model, the reliability of the results will be affected. The reason for the inclusive associations between the economic, social, cultural, policy, and physical environmental factors and SRB may be the failure to use the panel data and spatial econometric model. Thus, this paper aimed to examine the impact of economic, social, cultural, policy, and physical environmental factors on SRB using the dynamic spatial Durbin model (hereafter SDM) with considering the spatial dependence of SRB and its influencing environmental factors, and the time-lagged SRB.

## 3. Data and Methods

### 3.1. Methods

#### 3.1.1. Specification of Dynamic Spatial Durbin Model

The dynamic SDM mainly comprises three components: the time-lag of dependent variables, endogenous interaction effects, and exogenous interaction effects in the model. Referring to Elhorst [58,59], the specification of the model is as follows:

(1) $$Y_{t}=\tau Y_{t-1}+\rho WY_{t}+X_{t}\beta +WX_{t}\theta +\mu +\alpha_{t}+V_{t}$$

where *Yt* denotes an *N* × 1 column vector of the dependent variable, representing the SRB in *i* (*i* = 1, …, 31) province at *t* (*t* = 1, …, *t*); *Yt* − 1 represents the first-order time lag of SRB; *Xt* represents an *N × K* matrix of explanatory variables; *W* represents the *N × N* non-negative spatial weight matrix, describing the spatial connectivity of each unit. *WYt* represents the endogenous interaction effect, referring to the mutual influence of the dependent variables of each province through the spatial weight matrix. *WXt* represents the exogenous interaction effect, referring to the influence of independent variables of a province on SRB in other provinces through the spatial weight matrix. The parameters *τ, ρ, β, θ* are coefficients of the dependent variable’s first-order time lag *Yt* − 1, endogenous interaction effect *WYt*, explanatory variable and exogenous interaction effect *WXt*, respectively. *μ* is a *N* × 1 vector, used to control all variables that change with the province but not with time, called spatial-specific effects; *αt* is *N* × 1 vector, used to control all variables that do not change with the province, but change with time, called time-specific effects. Some studies have pointed out that it is more reasonable to use fixed effect models when using complete data at the national level [58]. Therefore, we chose time- and spatial-specific fixed effects models. The error term, *Vt*, is an *N* × 1 vector, containing independent and identically distributed error terms with a mean of 0 and a variance σ^2^.

#### 3.1.2. Direct and Indirect Effects

Due to the endogenous interaction effect *WYt* of SDM, the influence of a particular explanatory variable on the dependent variable at a time of this unit will also act on the dependent variable of that unit through the endogenous interaction effect (that is, “feedback effects”), meaning *β* in Equation (1) cannot truly reflect the influence of a specific explanatory variable on the dependent variable of this unit. Therefore, it is necessary to decompose the estimation results of the SDM model into direct effects and indirect effects (also called spillover effects) [60,61]. According to Elhorst [59], Equation (1) can be written as:

(2) $$Y_{t}={\left(I-\rho W\right)}^{-1}\tau Y_{t-1}+{\left(I-\rho W\right)}^{-1}\left(X_{t}\beta +WX_{t}\theta +\mu +\alpha_{t}+V_{t}\right)$$

The short-term effect of *K*-th explanatory variable of X in unit 1 up to unit N at time t on the dependent variable of all other units is:

(3) $${\left[\frac{\partial E\left(Y\right)}{\partial X_{1K}}\;\ldots \;\frac{\partial E\left(Y\right)}{\partial X_{NK}}\right]}_{t}={\left(I-\rho W\right)}^{-1}\left[\beta_{K}I_{N}+\theta_{K}W\right]$$

Equally, the long-term effects can be given by:

(4) $${\left[\frac{\partial E\left(Y\right)}{\partial X_{1K}}\;\ldots \;\frac{\partial E\left(Y\right)}{\partial X_{NK}}\right]}_{t}={\left[\left(1-\tau \right)I-\rho W\right]}^{-1}\left[\beta_{K}I_{N}+\theta_{K}W\right]$$

Among them, the direct effect is the average of the sum of the diagonal elements of the matrix of Equation (3) or Equation (4), which represents the average impact of a unit change of the *K*-th explanatory variable in a specific region on the dependent variable in the region. The indirect effect is the average of the row sums of the nondiagonal elements of the matrix of Equation (3) or Equation (4), which represents the average influence of a unit of the *K*th explanatory variable in a particular region on the dependent variable in all surrounding regions.

### 3.2. Data

In this paper, we adopted the data from 31 provinces in mainland China in 1982, 1990, 1995, 2000, 2005, 2010, and 2015 to examine the factors influencing the SRB from a spatial perspective. The SRB of each province is derived from the census or the one percent population sample surveys of the corresponding year [1,2,3,4,62,63,64]. Other data were from corresponding China Statistical Yearbooks [65], Almanac of China’s Population [66], China Population Statistics Yearbooks (renamed China Population and Employment Statistics Yearbook after 2007) [67], China Meteorological Yearbook [68], China Statistical Yearbook on Environment [69], and the census or the one percent population sample surveys [1,2,3,4,62,63,64].

There is considerable controversy regarding the data quality of China’s SRB. The National Health and Family Planning Commission is responsible for the implementation and monitoring of family planning policies and therefore tasked with the duty of recording annual SRB. However, since SRB is a performance evaluation indicator for local family planning departments, family planning cadres at all levels often manipulated data to limit the SRB within the scope of the evaluation requirements [70]. The National Bureau of Statistics is the authoritative statistical agency responsible for censuses and population sample surveys. Many scholars have evaluated the quality of SRB published by the National Bureau of Statistics [71,72] and found underreporting in the younger group of these data. Some studies believe that there was underreporting of female infants in the census data, so the actual SRB was lower than that published in the census [72,73], while others believe that underreporting was dominated by male births, and the actual SRB was higher than was reflected in the census [74]. For this study, we relied on the SRB data published by the Statistics Bureau without adjustment.

### 3.3. Measurements

*Dependent variable, SRB*. The dependent variable in this paper is the SRB, which represents the number of live male births corresponding to 100 live female births.

*Economic development, GDP*. Referring to previous research [13,21], we also used GDP per capita (logarithm) to represent economic development, and examined the relationship between the economic development level of a region and SRB.

*Urbanization level, URB*. Consistent with previous research [15,21], we used the urbanization rate to examine the relationship between urbanization level and SRB.

*Education level, EDU*. Some studies use average educational attainment or women’s average years of schooling to reflect the education level in a region [21]. Limited by the availability of data, it is also considered that high school education is a critical point to change gender ideology [13,21,37]. This article uses the proportion of the population with high school and above education levels to reflect the overall education level of a region.

*Family planning policies, STE*. For family planning policies, previous studies have measured the severity of Chinese family planning policies in various provinces with indicators like the total fertility rate [51] and policy fertility rate [21]. Some studies use fines for violating the family planning policies to represent the intensity of family planning policies [75,76]. Given the quality and availability of data, we selected the proportion of sterilized persons in birth control surgery to indirectly reflect the intensity of family planning policies.

*Air quality, SUL*. The strong dependence on coal as an energy in China has led to an increase in pollutants such as sulfur dioxide in the air. As one of the most common air pollutants and the main component of acid rain, sulfur dioxide has a serious impact on human health [77]. With reference to previous studies [41], we use per capita sulfur dioxide emissions to measure air quality.

*Ambient temperatures, TEM.* In line with previous studies [18,78], we used the annual average temperatures to measure ambient temperatures.

The definitions and descriptive statistics of variables are presented in Table 1.

### 3.4. Analytic Strategy

First of all, we calculated the global Moran’s I index of SRB and its influencing factors since 1982. Secondly, for comparison, we used traditional panel analysis methods to examine the relationship between SRB and explanatory variables without considering spatial dependence. Thirdly, we regressed with spatial error model (SEM), spatial autoregressive model (SAR), and spatial Durbin model (SDM) separately and showed the applicability of SDM in this paper. Finally, we fitted dynamic SDM and examined the effects of various explanatory variables on SRB through direct and indirect effects. We used the first-order queen contiguity spatial weight matrix in this study.

Spatial econometric models cannot be estimated if there are missing data, so we adopted multiple imputation method to impute missing values. Hainan Province, established as a province in 1988, was under Guangdong Province in 1982, and Chongqing City was under Sichuan in 1995. For some missing data in these regions, the data of Guangdong Province and Sichuan Province for the corresponding years were used. The models in this article were estimated using the *xsmle* command in Stata™ Version 13.1 software (StataCorp, College Station, TX, USA).

## 4. Results

### 4.1. Descriptive Analysis

Table 1 presents the definition and descriptive statistics of variables. Table 2 shows the global Moran’s I index of SRB and each explanatory variable, indicating that there is a spatial dependence between SRB and each explanatory variable. As a result, spatial clustering effects needed to be considered in the analysis.

### 4.2. Results of OLS and Spatial Models

Table 3 shows the regression results. Firstly, the OLS estimation results show that STE had a significant effect on SRB. That is, the higher the proportion of people performing sterilization, the higher the SRB in the region. GDP, URB, EDU, SUL, and TEM had no statistically significant effect on SRB.

Secondly, in the results of the three models SEM, SAR, and SDM, the direction of the influence of four explanatory variables on SRB did not change, but the statistical significance changed (see the main panel in Table 3). The three models are intercorrelated, in Equation (1), if θ = 0, SDM could be simplified to SAR; if θ + ρβ = 0, SDM could be simplified to SEM [50,79]. Referring to the test method in Belotti et al. [80], the results show that SDM cannot be simplified to SAR (F = 2.35, *p* = 0.029) or SEM (F = 2.86, *p* = 0.009).

Furthermore, previous studies have suggested that there may be an inverse U-shaped relationship between explanatory variables and SRB [12,13]. We included the square of explanatory variables in SDM, but the results were not significant (results were not given). We also compared fixed effect with random effect in SDM using the Hausman test, and the results supported the fixed effects (χ^2^ = 19.13, *p* = 0.004).

Finally, we estimated the dynamic SDM by introducing the first-order time lag of SRB in the SDM. The time-lagged SRB was significantly positive, indicating an inertia in the change of SRB, and other potential factors also had a significant positive effect on the change of SRB. The spatial autoregressive coefficients (*Rho* in Table 3) show that SDM overestimated the degree of spatial dependence (*Rho* = 0.355 in SDM vs. *Rho* = 0.334 in Dynamic-SDM). The reason may be that the effects of other factors not included in the model on SRB were generally classified as spatial dependence. Therefore, the following analysis was based on the results of Dynamic-SDM.

### 4.3. Average Direct and Indirect Effects of Dynamic-SDM

Lesage and Pace [50] pointed out that in spatial econometric models, the spatial dependence makes the coefficients of independent variables no longer appropriate for measuring the influence and statistical significance of the variable, rather, the effects of independent variables on the dependent variable should be decomposed into direct and indirect effects, and then the model could be explained. Table 4 presents the short-term and long-term direct effects, indirect effects, and total effects of Dynamic-SDM. Since the analysis was based on data observed over five-year time intervals, the short-term effects did not differ significantly from the long-term effects. For this reason, we only explained the results based on long-term effects.

First, the direct effects of GDP were not significant, but the indirect effects were significantly positive, indicating that the economic development of a region would increase the SRB in other regions.

Second, the urbanization level had a significantly negative direct effect and positive indirect effect. The negative direct effects indicated that the urbanization level in a region would significantly reduce the SRB in this region, and this is consistent with previous studies [15,21]. The positive indirect effect suggested that urbanization level in one region would lead to an increase in SRB in other regions, which was underexamined before.

Third, education level had a significant negative indirect effect, although the direct effect was not significant. The negative indirect effect showed that the improvement of the education level in one region would reduce the SRB in neighboring regions.

Fourth, family planning policies had a significant positive direct effect, while the indirect effect was not significant. The positive direct effect indicated that the stricter the family planning policies in a region, the higher SRB in this region, as consistent with previous research [39,40].

Finally, both the per capita sulfur dioxide emissions and the annual average temperatures did not have significant direct or indirect effects on SRB.

Comparison between the direct effects, indirect effects, and total effects of the explanatory variables showed the following: for the direct effect, URB was the largest in magnitude, followed by STE; for the indirect effect, GDP had the greatest effect on SRB in the surrounding region, followed by EDU, and finally URB; for the total effect, GDP and EDU had a greater effect on SRB. Comparing the direct, indirect, and total effects of different variables, the increase in the economic and social development level (GDP per capita and urbanization rate) of a region would inhibit the increase in the local SRB, but it was not conducive to the governance of SRB in the surrounding regions. The improvement of people’s educational level in a region not only benefited the governance of the SRB in the region, but also had an inhibitory effect on the SRB in surrounding regions; and the family planning policies would only affect the local SRB.

## 5. Discussion

Based on China’s census and population sample survey data since 1982, we examined the effect of economic development, urbanization level, education level, and family planning policies on SRB with the dynamic spatial Durbin model. We found that GDP per capita had a positive indirect effect on SRB; the urbanization rate had a negative direct and positive indirect effect; the proportion of people with high school education and above had a negative direct and indirect effect; the proportion of people performing sterilization had a positive direct effect.

Economic and social development is conducive to the governance of SRB in this region, but will aggravate the imbalance of SRB in the neighboring regions. International experience shows that as countries approach high levels of development, modernization, and urbanization, the value of sons and daughters to parents tends to become more equal [28]. With social and economic transformation, son preference will decline [28,81]. China’s labor force flows from economically underdeveloped regions to more economically developed regions, from rural regions to towns and nonagricultural industries [21,55]. Therefore, economic and social development in one region will attract population migration from neighboring regions. Urbanization has created opportunities for migrant workers to improve their knowledge and accept new fertility ideology, which has gradually weakened son preferences [21,32]. Additionally, as a large number of female migrants move to cities for work, they become economically independent and more decisive in family, which also reduces the possibility of sex-selective abortion [33]. However, with the out-migration, the proportion of people with relatively low education level, traditional fertility ideology and fertility behavior will increase in the original regions [82], which may relatively increase the SRB in those regions.

Educational improvement is beneficial to the governance of SRB in neighboring regions. Previous studies have suggested that higher education development appears to spatially cluster and spillover, and the rapid growth of education in one region will drive the development of education in neighboring regions [56]. With the educational improvement in neighboring regions, SRB in the surrounding regions also declines. Therefore, the educational improvement in one region can facilitate to normalize the SRB in surrounding regions.

The effect of the family planning policies on SRB is limited to the local region, and there is no spatial spillover effect. As a national policy, China’s family planning policy was provincially localized [20]. As people are required to comply with the provincial family planning policies of where their household is registered, even when they migrate to other provinces, they are still limited to the number of births set by the province where their household is registered. Though there were several categories in terms of the birth number permitted, people rarely migrated with their household registration to other provinces simply because of different family planning policies. Although people may leave their homes to avert family planning [83], this escape was confined to a short distance. As births should be registered where the household registration of this family is, it is not likely to produce a spatial spillover effect.

However, the associations between physical environments and SRB were nonsignificant in this study. Consistent with previous studies [44,45,49], this paper did not find that air quality and ambient temperatures have a significant influence on SRB. The reason may be that it would need a long time for the physical environments to have an impact on SRB, while the time frame in this article was only about 30 years.

There are some limitations in this paper. First, the correlation between the explanatory variables may affect the results. The explanatory variables in this paper include GDP per capita, urbanization level, education level, and family planning policies. These synthetic indicators are interrelated. Although the VIF (Variance inflation factor) value of each explanatory variable is less than 10, it may still have a certain impact on the statistical significance of the results. Second, there may be some problems with the data quality used in this article. Despite China’s SRB data being controversial, we used directly the data released by the national bureau of statistics without adjustment as there is no other reliable data to be used as a benchmark against which to adjust the census data. Finally, due to the unavailability of data, we used province-level data, which may be a large unit for spatial analysis.

## 6. Conclusions

In conclusion, our findings suggest that the influence of spatial dependence should be considered when studying the changes in China’s SRB. The changes in SRB are the result of the interaction of social, economic, cultural, policy, and physical environment. Although China’s 31 provinces have their own unique socioeconomic, cultural, policy, and natural environmental characteristics, population movements and other difficult-to-observe factors between neighboring provinces will affect the SRB in neighboring areas. Therefore, when governing the SRB, it is necessary to comprehensively consider the mutual influence of neighboring areas and formulate reasonable intervention policies through education policies and other polices under the framework of sustainable development [84].

## Figures and Tables

**Table 1:** Descriptive statistics for SRB (sex ratio at birth) and explanatory variables.

Variable	Definition	Mean	SD	Observation
*Dependent variable*				
SRB	The ratio of live male births to 100 live female births	114.172	8.030	217
*Explanatory variables*				
GDP	Natural log of gross domestic product per capita	8.810	1.555	217
URB	Percentage of people live in the cities or towns	39.913	18.753	217
EDU	Percentage of people with high school education or above	18.844	10.486	186
STE	Percentage of people sterilized during birth control	38.432	19.816	183
SUL	Per capita sulfur dioxide emissions	17.632	11.601	186
TEM	Annual average temperatures	14.140	5.080	186

Notes: SRB denotes Sex ratio at birth; GDP denotes Economic development; URB denotes Urbanization level; EDU denotes Education level; STE denotes Family planning policies; SUL denotes Air quality; TEM denotes Ambient temperatures; SD denotes Standard deviation; VIF denotes Variance inflation factor; The largest VIF value of explanatory variables was less than 10.

**Table 2:** Global Moran’s Index of SRB and Explanatory variables since 1982.

Year	SRB	GDP	URB	EDU	STE	SUL	TEM
1982	0.362 ***	0.173 **	0.348 ***	0.279 ***	-	-	-
1990	0.105	0.261 **	0.307 ***	0.291 ***	0.221 **	0.266 ***	0.758 ***
1995	0.361 ***	0.304 ***	0.236 **	-	0.256 **	0.229 **	0.727 ***
2000	0.520 ***	0.365 ***	0.310 ***	0.328 ***	0.310 ***	0.162 *	0.723 ***
2005	0.253 ***	0.320 ***	0.380 ***	0.318 ***	0.304 ***	0.233 **	0.739 ***
2010	0.499 ***	0.438 ***	0.403 ***	0.327 ***	0.303 ***	0.256 **	0.754 ***
2015	0.004	0.395 ***	0.406 ***	0.301 ***	0.263 ***	0.327 ***	0.723 ***

Notes: SRB denotes Sex ratio at birth; GDP denotes Economic development; URB denotes Urbanization level; EDU denotes Education level; STE denotes Family planning policies; SUL denotes Air quality; TEM denotes Ambient temperatures; “-” refers to a missing value; *** *p* < 0.01, ** *p* < 0.05, * *p* < 0.1.

**Table 3:** Estimation of the regression of SRB on OLS and spatial panel models from 1982–2015.

Variable	OLS	SEM	SAR	SDM	Dynamic-SDM
*SRB lagged in time*					
Yt − 1					0.190 **
*Main*					
GDP	0.978	4.710 ***	3.435 ***	0.536	−1.449
URB	−0.075	−0.193 *	−0.092	−0.192 *	−0.342 ***
EDU	−0.245	−0.197	−0.240 *	−0.169	−0.140
STE	0.157 ***	0.131 **	0.134 ***	0.121 **	0.167 **
SUL	0.032	0.007	0.013	0.009	0.085
TEM	0.243	0.125	0.080	0.065	0.450
*Spatial effect*					
Lambda		0.473 ***			
Rho			0.442 ***	0.355 ***	0.334 ***
W*GDP				2.813	4.454 *
W*URB				0.390 ***	0.488 ***
W*EDU				−0.479 *	−0.461 *
W*STE				0.043	0.022
W*SUL				0.115	0.061
W*TEM				−0.010	−0.904

Notes: SRB denotes Sex ratio at birth; GDP denotes Economic development; URB denotes Urbanization level; EDU denotes Education level; STE denotes Family planning policies; SUL denotes Air quality; TEM denotes Ambient temperatures; W denotes Spatial weight matrix; OLS denotes Ordinary least squares; SEM denotes Spatial error model; SAR denotes Spatial autoregressive model; SDM denotes Spatial Durbin model; All models are fixed with spatial and time effects except the Dynamic-SDM; *** *p* < 0.01, ** *p* < 0.05, * *p* < 0.1.

**Table 4:** The direct, indirect, and total effects of Dynamic-SDM in the short- and long-term effects.

Variable	Short-term effects			Long-term effects		
	Direct	Indirect	Total	Direct	Indirect	Total
GDP	−1.118	5.466 **	4.348 **	−1.276	7.349 **	6.073 **
URB	−0.306 ***	0.530 ***	0.224	−0.370 ***	0.685 **	0.315
EDU	−0.187	−0.698 **	−0.885 **	−0.249	−0.993 **	−1.241 **
STE	0.176 ***	0.104	0.280 *	0.221 ***	0.172	0.393
SUL	0.092	0.131	0.223	0.118	0.199	0.316
TEM	0.404	−1.088	−0.684	0.477	−1.454	−0.977

Notes: GDP denotes Economic development; URB denotes Urbanization level; EDU denotes Education level; STE denotes Family planning policies; SUL denotes Air quality; TEM denotes Ambient temperatures; SDM denotes Spatial Durbin model; *** *p* < 0.01, ** *p* < 0.05, * *p* < 0.1.
